# Reduced Life- and Healthspan in Mice Carrying a Mono-Allelic *BubR1* MVA Mutation

**DOI:** 10.1371/journal.pgen.1003138

**Published:** 2012-12-27

**Authors:** Tobias Wijshake, Liviu A. Malureanu, Darren J. Baker, Karthik B. Jeganathan, Bart van de Sluis, Jan M. van Deursen

**Affiliations:** 1 Department of Pediatric and Adolescent Medicine, Mayo Clinic, Rochester, Minnesota, United States of America; 2 Department of Pathology and Medical Biology, University Medical Center Groningen, Groningen, The Netherlands; 3 Department of Biochemistry and Molecular Biology, Mayo Clinic, Rochester, Minnesota, United States of America; The University of Texas Health Science Center at San Antonio, United States of America

## Abstract

Mosaic Variegated Aneuploidy (MVA) syndrome is a rare autosomal recessive disorder characterized by inaccurate chromosome segregation and high rates of near-diploid aneuploidy. Children with MVA syndrome die at an early age, are cancer prone, and have progeroid features like facial dysmorphisms, short stature, and cataracts. The majority of MVA cases are linked to mutations in *BUBR1*, a mitotic checkpoint gene required for proper chromosome segregation. Affected patients either have bi-allelic *BUBR1* mutations, with one allele harboring a missense mutation and the other a nonsense mutation, or mono-allelic *BUBR1* mutations combined with allelic variants that yield low amounts of wild-type BubR1 protein. Parents of MVA patients that carry single allele mutations have mild mitotic defects, but whether they are at risk for any of the pathologies associated with MVA syndrome is unknown. To address this, we engineered a mouse model for the nonsense mutation 2211insGTTA (referred to as GTTA) found in MVA patients with bi-allelic *BUBR1* mutations. Here we report that both the median and maximum lifespans of the resulting *BubR1* ^+/GTTA^ mice are significantly reduced. Furthermore, *BubR1* ^+/GTTA^ mice develop several aging-related phenotypes at an accelerated rate, including cataract formation, lordokyphosis, skeletal muscle wasting, impaired exercise ability, and fat loss. *BubR1* ^+/GTTA^ mice develop mild aneuploidies and show enhanced growth of carcinogen-induced tumors. Collectively, these data demonstrate that the *BUBR1* GTTA mutation compromises longevity and healthspan, raising the interesting possibility that mono-allelic changes in *BUBR1* might contribute to differences in aging rates in the general population.

## Author Summary

Aging is the main risk factor for the majority of chronic diseases and the leading cause of death and disability in humans. Humans age at different rates, but the molecular genetic basis underlying this phenomenon remains largely unknown. Efforts to understand how we age have focused on genetic changes that extend lifespan or underlie progeroid disorders. One potential progeroid disorder, MVA syndrome, has been associated with mutations in the mitotic regulator *BUBR1*. Although MVA syndrome is rare due to its recessive nature, individuals carrying heterozygous *BUBR1* mutations associated with MVA would be much more prevalent. However, whether such carriers are asymptomatic or at risk of developing aspects of MVA syndrome later in life is unknown. To investigate this, we engineered mice to carry an analogous mutation to the human MVA *BUBR1* nonsense mutation 2211insGTTA. We find that these mice have a reduced lifespan and develop several age-related phenotypes at an accelerated rate. These findings suggest that bi-allelic integrity of *BUBR1* is a key determinant of healthspan and longevity, and provide a conceptual framework for elucidating differences in aging rates among humans.

## Introduction

Separation of duplicated chromosomes during mitosis is an intricate biological process whose molecular basis is incompletely understood. Inaccurate segregation of whole chromosomes results in numerical chromosome aberrations, referred to as aneuploidy [1]. Human aneuploidy is intimately associated with developmental defects and disease pathology [2]. For example, aneuploidy in gametes is a known cause of infertility, miscarriages and congenital birth defects [3], while somatic cell aneuploidy is a hallmark of cancer, with evidence mounting that aneuploidy can promote neoplastic transformation [4]–[6]. To safeguard against chromosome missegregation, eukaryotic organisms have developed cellular surveillance systems including the mitotic checkpoint (or spindle assembly checkpoint) and the attachment error correction machinery [7]. The mitotic checkpoint is a multi-protein network that inhibits sister chromatid separation until all chromosomes are properly attached to the mitotic spindle [8]. One of the core components of this checkpoint is BubR1, a modular protein that acts to inhibit the activity of the large multi-protein E3 ubiquitin ligase known as the anaphase promoting complex/cyclosome (APC/C), by binding to the co-activating subunit Cdc20 [9]. Once all chromosomes have achieved bi-orientation, BubR1 dissociates from Cdc20 leading to the polyubiquitination of securin and cyclin B1, two inhibitors of separase, a protease that initiates anaphase by cleaving cohesin rings that physically join duplicated sister chromosomes together. BubR1 not only promotes accurate chromosome segregation in its role as a Cdc20 inhibitor, but also acts to stabilize microtubule-kinetochore attachments [10].

*BUBR1* mutations have been identified in various human malignancies, including gastrointestinal cancers [11]–[14], and in a rare human hereditary condition called mosaic variegated aneuploidy (MVA) syndrome, in which high rates of chromosome missegregation lead to systemic aneuploidy, typically involving more than 25% of cells [15], [16]. MVA syndrome has clinically heterogeneic features, including growth deficiency (with prenatal onset), mental retardation, microcephaly, facial dysmorphisms, cataracts and other eye abnormalities, short lifespan, and increased risk for childhood cancers such as rhabdomyosarcoma, Wilms' tumor and leukemia [17]. MVA patients with *BUBR1* mutations fall into two groups: bi-allelic and mono-allelic mutations. Patients with bi-allelic mutations carry an allele that results in premature truncation of BubR1 protein or an absent transcript, and an allele with a missense mutation often located within the kinase domain [15]. Patients with mono-allelic mutations have either a nonsense or missense mutation combined with a non-mutated allelic *BUBR1* variant that expresses low amounts of wildtype BubR1 protein [16], [17]. BubR1 protein levels are usually very low in patients with *BUBR1* mutations, even in those with a missense mutation, largely because mutant BubR1 proteins produced by these alleles tend to be quite unstable [18].

Gene knockout studies in mice support the idea of a causal relationship between BubR1 insufficiency and tumorigenesis. Although homozygous *BubR1* knockouts die as pre-implantation stage embryos, heterozygous knockouts are viable and show increased tumor formation when challenged with a carcinogen [19]–[21]. BubR1 depletion beyond the level of heterozygous knockout mice was achieved by the use of knockout (*BubR1* ^−^) and hypomorphic (*BubR1* ^H^) alleles [21]. Mice with one hypomorphic and one knockout allele (*BubR1* ^−/H^ mice) express about 4% of normal BubR1 protein levels and die at birth from respiratory failure [21]. However, mice with two *BubR1* hypomorphic alleles (*BubR1* ^H/H^ mice) that produce around 10% of normal BubR1 protein levels are viable. Like MVA patients, these mice show systemic near-diploid aneuploidy, premature chromosome separation (PCS) and are susceptible to tumorigenesis [21], [22]. However, unlike other mouse models for aneuploidy that have similar features [1], *BubR1* ^H/H^ mice have a very short lifespan and develop several progeroid phenotypes at a very young age (within 3 to 5 months), including growth retardation (dwarfism), facial dysmorphisms, cataracts, muscle wasting, lordokyphosis (rearward curvature of the spine), fat loss, and cardiac arrhythmias [21], [23]. This, together with observations that BubR1 protein levels decrease during natural aging in various mouse tissues, raises the possibility that *BubR1* is an important regulator of aging [21], [24].

MVA syndrome has been documented as a hereditary cancer syndrome [15]–[18], [25]–[27], but could potentially be classified also as a progeroid syndrome based on its phenotypic resemblance to *BubR1* progeroid mice, which includes short lifespan, dwarfism, facial dysmorphisms, and cataract formation. However, whether MVA patients have additional age-related phenotypes observed in *BubR1* hypomorphic mice such as fat loss, muscle wasting and cardiac arrhythmias is a key open question that has been difficult to address, largely because MVA syndrome is very rare and because most patient die very young. While patients with the MVA disorder are rare, heterozygous carriers of *BUBR1* mutant alleles are expected to be much more prevalent in the general population. If these mutations were to affect healthspan or longevity, or both, this might provide a genetic basis for why certain people develop particular age-related traits at faster rates. Little is known about the health status of parents of MVA patients. They seem to have a minor mitotic phenotype as evidenced by their predisposition to PCS, but whether they are at risk for any of the pathologies associated with MVA syndrome or progeria is unknown [28]–[30]. To address this question and to better understand the relationship between MVA syndrome and progeria, we engineered mice to carry the human MVA *BUBR1* nonsense mutation 2211insGTTA [15]. We demonstrate that mice harboring this heterozygous *BubR1* MVA mutation have a reduced lifespan and exhibit acceleration of various early age-related features. In addition, reduced BubR1 protein levels in *BubR1* ^+/GTTA^ mice results in mild aneuploidy and increases carcinogen-induced tumor growth. These findings suggest that mono-allelic *BUBR1* mutations might contribute to accelerated aging and reduced longevity, further supporting the idea that MVA syndrome is a progeria-like syndrome. Additionally, we provide important experimental evidence for the longstanding concept that variations in select genes accelerate the rate of age-related deterioration of certain tissues and organs.

## Results

### Generation of a mouse model for MVA *BubR1* mutation 2211insGTTA

**Figure 1:**
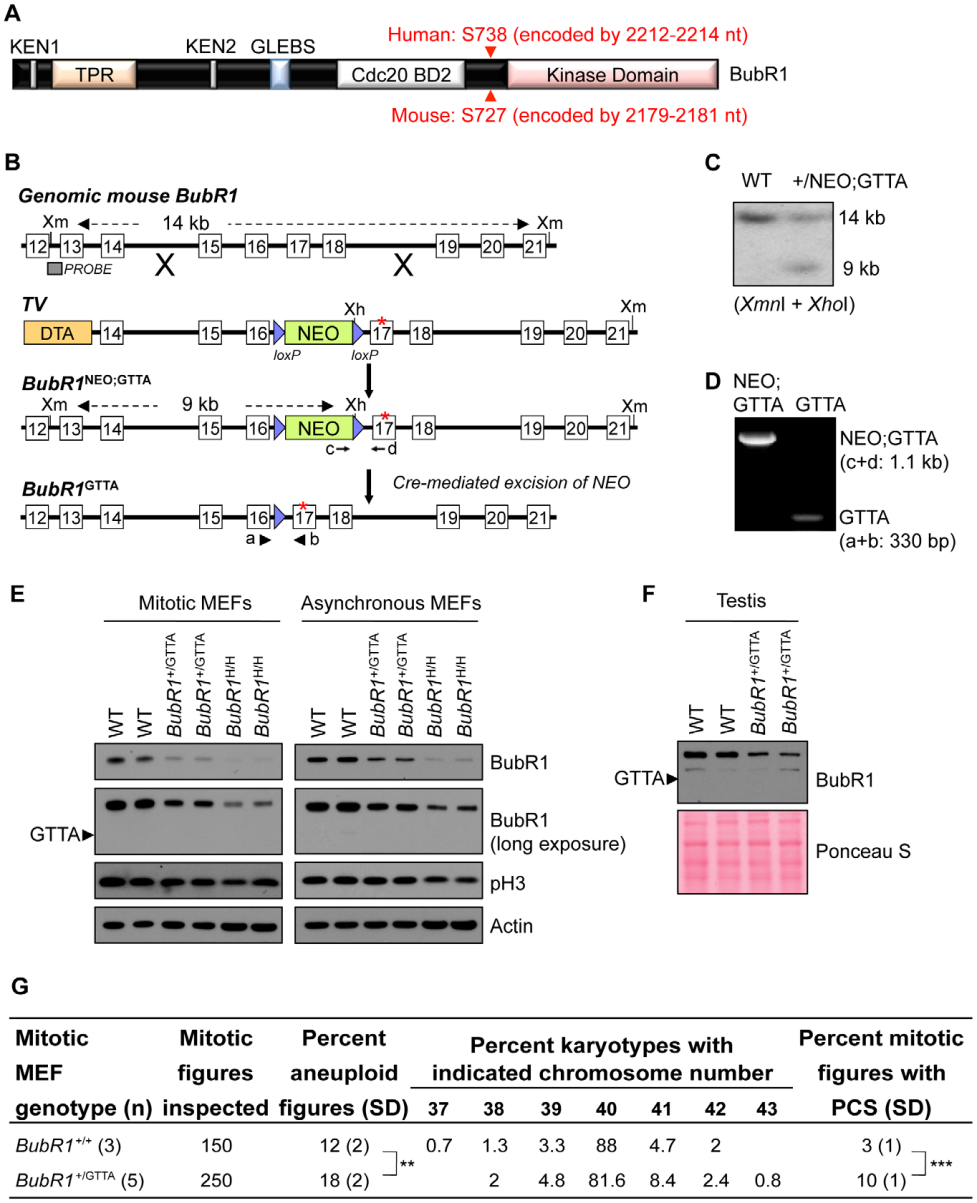
*BubR1* ^+/GTTA^ mice are viable and mimic their human counterpart. (A) Schematic representation of BubR1 protein. Functional domains are indicated: KEN, destruction box-motifs implicated in APC/C^Cdc20^ inhibition (also referred to as Cdc20 BD1); TPR, tetratricopeptide motif for binding to Knl1; GLEBS, motif for Bub3 binding and kinetochore localization; Cdc20 BD2, C-terminal Cdc20 binding domain, Kinase domain, protein kinase domain homology domain. Red arrowheads indicate human and mouse GTTA insertion sites: the first amino acid of BubR1 impacted by the insertion is indicated in red font. (B) Gene targeting strategy to generate *BubR1* ^+/GTTA^ mice. Shown are: the genomic mouse *BubR1* locus spanning exon 12 to 21 (top); the targeting vector (TV) with the GTTA insertion in exon 17 (asterisk), *loxP* sites (blue triangles); the BubR1 locus after targeted recombination; and the final *BubR1* ^+/GTTA^ locus after protamine-Cre mediated recombination mice to excise the NEO cassette and produce *BubR1* ^+/GTTA^ mice. The *Xmn*1 (*Xm*) and *Xho*1 (*Xh*) restriction sites, the Southern blot probe, wildtype and *BubR1* ^NEO;GTTA^ Southern blot fragments, and the 4 PCR primer sites (a–d) used for genotyping are indicated. (C) Southern blot containing *Xmn*1/*Xho*1 digested genomic DNA from two ES cell clones and probed with DNA fragment indicated in (B) showing the 14 kb and 9 kb fragments representing the wildtype and *BubR1* ^NEO;GTTA^ alleles, respectively. (D) PCR genotyping of two pups from a *BubR1* ^+/NEO:GTTA^ female crossed to a protamine-Cre male. One pup had the NEO cassette successfully removed to create a *BubR1* ^+/GTTA^ allele. (E) Western blots of mitotic and asynchronous MEF extracts probed for BubR1, the mitotic marker p-H3, and actin (loading control). (F) Western blot of testis extracts probed for BubR1. Ponceau S staining was used as loading control. (G) Karyotype analysis performed on wildtype and *BubR1* ^+/GTTA^ P5 MEFs. Fifty mitotic figures were counted per sample. PCS, premature chromatid separation. An unpaired t-test was used for statistical analysis. **P<0.01, ***P<0.001.

Human *BUBR1* encodes a modular 1052 amino acid serine/threonine protein kinase that is highly conserved among mammalian species (Figure 1A). Two of the four MVA patients with bi-allelic *BUBR1* mutations reported by Hanks and colleagues [15] carry a nonsense mutation referred to as 2211insGTTA that results in a truncated protein that lacks the kinase domain (Figure 1A). Because of its prevalence, we mimicked this mutation in mice to understand the potential physiological consequences of MVA mutations in a heterozygous state. Using homologous recombination in embryonic stem (ES) cells, we inserted a GTTA sequence in the murine *BubR1* gene at position 2178. This position corresponds to nucleotide 2211 of the human *BUBR1* transcript (Figure 1A and 1B). We injected correctly targeted ES clones into blastocysts and obtained chimeric mice. These chimeras successfully transmitted the mutated *BubR1* allele (referred to as *BubR1* ^NEO;GTTA^) to their offspring (Figure 1B and 1C). *BubR1* ^+/NEO;GTTA^ mice were crossed to protamine-Cre transgenic mice to remove the NEO gene cassette (Figure 1B and 1D). *BubR1* ^+/GTTA^ mice were obtained at the expected frequency and were overtly indistinguishable from control littermates (data not shown).

Western blot analysis demonstrated that mouse embryonic fibroblasts (MEFs) derived from *BubR1* ^+/GTTA^ mice had reduced amounts of wildtype BubR1 protein (Figure 1E). The level of reduction was similar to that observed in *BubR1* ^+/−^ MEFs (Figure 1E and Figure S1). The predicted 730 amino acid truncated protein encoded by the *BubR1* ^GTTA^ allele was undetectable, even after long exposure times (Figure 1E), which is consistent with recent observations in cultured skin fibroblast of an MVA patient carrying the *BUBR1* ^GTTA^ allele [18]. Western blot analysis of testis extracts from *BubR1* ^+/GTTA^ and wildtype mice confirmed the reduction of BubR1 in cultured MEFs (Figure 1F). One plausible explanation for the absence of *BubR1* ^GTTA^ encoded protein on western blots is that nonsense mutations tend to produce transcripts that are subject to non-sense mediated mRNA decay [13], [16], [18]. Alternatively, the truncated protein may be unstable and subject to rapid proteosomal degradation.

Patients with MVA syndrome show systemic near-diploid aneuploidies resulting from an inability to separate duplicated chromosomes with high accuracy during mitosis [15], [16], [29]. Although metaphase spreads from heterozygous carriers of MVA *BUBR1* mutations exhibit increased rates of PCS, it is unclear whether parents of MVA patients are subject to increased aneuploidization [15], [16], [26], [31]–[34]. To examine the impact of the *BubR1* ^GTTA^ allele on chromosome number integrity, we prepared metaphase spreads of passage 5 *BubR1* ^+/GTTA^ and wildtype MEFs and performed chromosome counts. Aneuploidy rates of wildtype MEFs were on average 12% (Figure 1G), which is consistent with rates reported in previous studies [35]–[37]. Aneuploidy rates in *BubR1* ^+/GTTA^ MEFs were significantly increased by 6%, although it should be noted that this escalation is relatively small compared to that previously observed in *BubR1* ^H/H^ MEFs [21] (Table S1). Consistent with this, *BubR1* ^H/H^ MEFs exhibited a more profound reduction in wildtype BubR1 protein levels than *BubR1* ^+/GTTA^ MEFs (Figure 1E and Table S1). The incidence of PCS was also significantly higher in *BubR1* ^+/GTTA^ MEFs than in wildtype MEFs (Figure 1G), but again not as high as in *BubR1* ^H/H^ MEFs [21]. Collectively, these data indicate that the *BubR1* ^GTTA^ allele that we created in mice faithfully mimics its human counterpart, and demonstrate that *BubR1* ^+/GTTA^ mice have relatively mild, but significant, mitotic phenotypes.

### Median and maximum lifespans of *BubR1* ^+/GTTA^ mice are reduced

**Figure 2:**
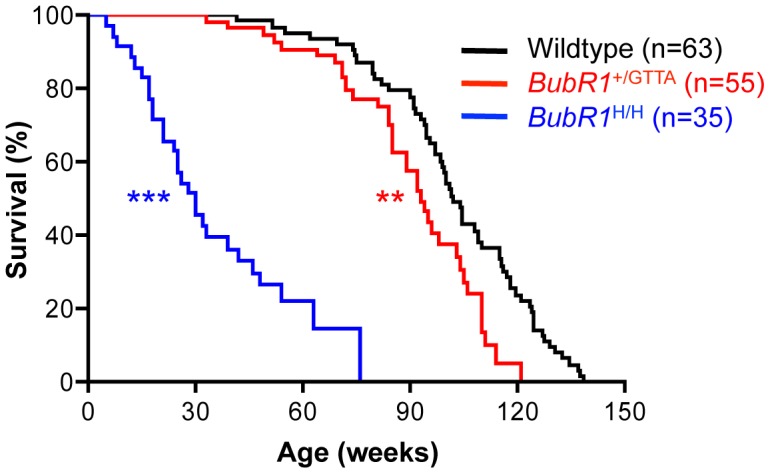
Lifespan of *BubR1* ^+/GTTA^ is reduced. Kaplan-Meier overall survival curves. Asterisks denote significance compared to wildtype mice using a log-rank test: **P<0.01 and ***P<0.001. The maximum lifespan was significantly decreased in *BubR1* ^+/GTTA^ compared to wildtype (*P* = 0.0008; two-sided Wang/Allison test referring to the ratio of mice alive per genotype at the 90^th^ percentile survival point) [70].

*BubR1* ^H/H^ mice exhibit various aging-related phenotypes by 3 to 4 months of age [21] (Table S1). *BubR1* ^+/GTTA^ mice, however, remained overtly indistinguishable from control littermates during this time period. To examine potential late life phenotypes associated with the GTTA mutation, we established large cohorts of *BubR1* ^+/GTTA^ and wildtype mice, which we monitored for signs of ill health and the development of overt age-related phenotypes. Kaplan-Meier overall survival curves of these cohorts showed that the GTTA mutation significantly reduces median and maximum lifespan, with *BubR1* ^+/GTTA^ mice having a median lifespan of 93 weeks compared to 102 weeks for wildtype mice (Figure 2). By comparison, *BubR1* ^H/H^ mice had a significantly shortened median survival of only 30 weeks (Figure 2).

*BubR1* ^H/H^ mice develop quite severe cardiac arrhythmias that are thought to be the primary cause of premature death of the animals [38] (Table S1). This prompted us to test whether reduced lifespan of *BubR1* ^+/GTTA^ mice might be due to cardiac problems. However, the frequency of cardiac arrhythmias in *BubR1* ^+/GTTA^ mice was not elevated (Figure S2A). Subsequent cardiac stress tolerance tests, in which a lethal dose of the β-adrenergic agonist isoproterenol was injected and the time to death measured [39], further indicated that the cardiac performance of *BubR1* ^+/GTTA^ mice is not compromised (Figure S2B).

### Muscle wasting and cataract formation are accelerated in *BubR1* ^+/GTTA^ mice

**Figure 3:**
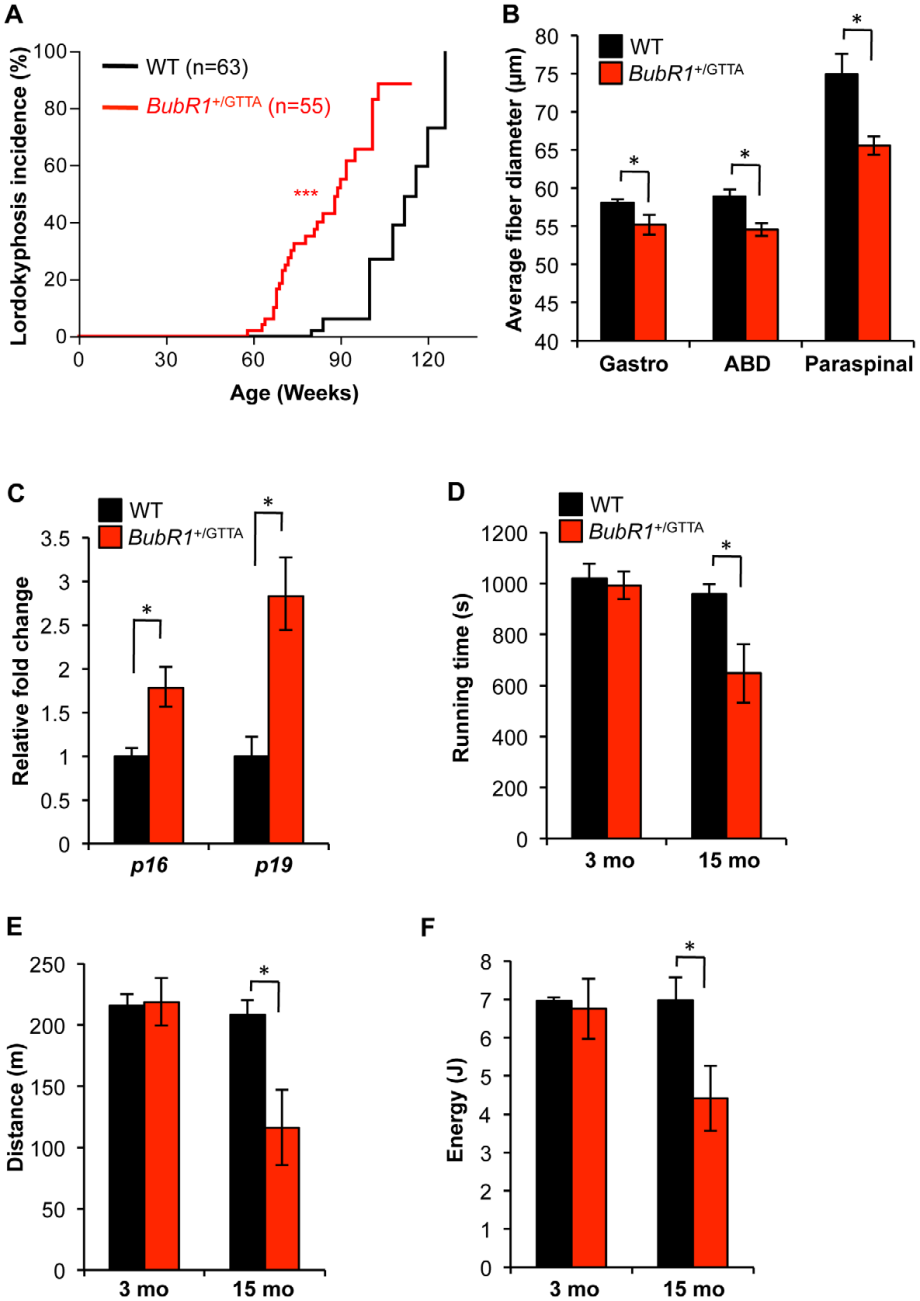
Accelerated deterioration of skeletal muscle in *BubR1* ^+/GTTA^ mice. (A) Incidence and latency of lordokyphosis. A log rank test was used for statistical analysis: ***P<0.001. (B) Mean fiber diameter of gastrocnemius (Gastro), abdominal (ABD), and paraspinal muscles of 15-month-old mice. (C) Expression of the senescence markers *p16* ^Ink4a^ and *p19* ^Arf^ in gastrocnemius muscles of 15 month-old mice analyzed by qRT-PCR. (D–F) Treadmill exercise ability of 3- and 15-month-old mice: presented are running time (D), distance travelled (E), and workload (F) performed. Error bars represent SEM. Males mice were used for all analysis in D–F: wildtype 3 months, n = 3 males; *BubR1* ^+/GTTA^ 3 months, n = 5 males, wildtype and *BubR1* ^+/GTTA^ 15 months, n = 4 males each. An unpaired t-test was used for statistical analyses in (B–F): *P<0.05.

A prominent phenotype of *BubR1* hypomorphic mice is lordokyphosis [21]. The underlying condition here is skeletal muscle deterioration rather than osteoporosis [23]. *BubR1* ^+/GTTA^ and wildtype mice in our cohorts were biweekly monitored for this phenotype. Wildtype mice are known to develop lordokyphosis as part of their normal aging process [40], a finding commonly attributed to a combination of muscle wasting and osteoporosis [41], [42]. While mice in both our cohorts indeed developed lordokyphosis with aging, the median onset of this age-related phenotype was markedly accelerated in *BubR1* ^+/GTTA^ mice (89 weeks versus 116 weeks; Figure 3A).

To determine whether this acceleration might be due to early muscle degeneration, we sacrificed 15-month old *BubR1* ^+/GTTA^ and wildtype mice and performed muscle fiber diameter measurements on cross sections of the gastrocnemius, paraspinal and abdominal muscles. As shown in Figure 3B, average muscle fiber diameters of *BubR1* ^+/GTTA^ mice were significantly reduced in all three muscle groups. No such reductions were observed in 3-month-old *BubR1* ^+/GTTA^ mice (Figure S3). Using qRT-PCR we analyzed the gastrocnemius of aged *BubR1* ^+/GTTA^ and wildtype mice for expression of *p16* ^Ink4a^ and *p19* ^Arf^ and found that both senescence markers were expressed at elevated levels [23], [38], suggesting that senescent cells might contribute to accelerated muscle degeneration in *BubR1* ^+/GTTA^ mice (Figure 3C). Furthermore, 15-month old *BubR1* ^+/GTTA^ mice had similar bone mineral densities and contents as wildtype mice as measured by dual energy x-ray absorptiometry (DEXA; Figure S4), demonstrating that the early kyphosis is not due to accelerated osteoporosis.

To assess whether early muscle wasting resulted in decreased muscle function, we performed treadmill exercise tests on *BubR1* ^+/GTTA^ and wildtype mice at various ages [38], [43]. As shown in Figure 3D–3F, wildtype mice showed similar exercise ability at 3 and 15 months of age. However, while *BubR1* ^+/GTTA^ mice showed normal exercise ability at 3 months, the duration of exercise, distance travelled and overall amount of work performed were all significantly decreased at 15 months.

**Figure 4:**
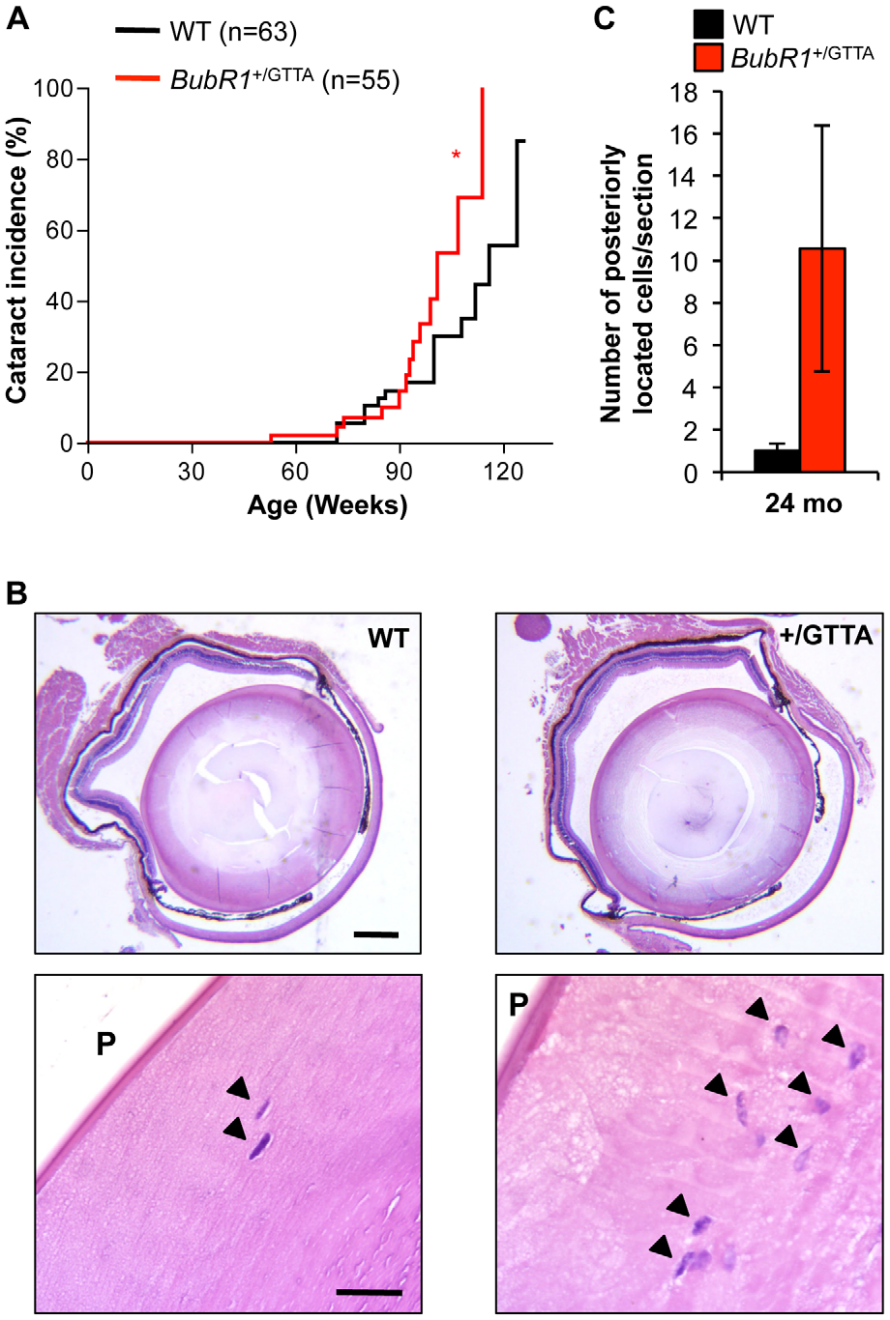
Cataract formation is accelerated in *BubR1* ^+/GTTA^ mice. (A) Incidence and onset of cataracts in *BubR1* ^+/GTTA^ and wildtype mice. Eyes were dilated and screened for cataracts using a slit light. A log rank test was used for statistical analysis: *P<0.05. (B) Representative cross sections of cataractous lenses stained with hematoxylin and eosin. Note that the *BubR1* ^+/GTTA^ lens section contains more posteriorly located (arrowheads). Scale bars represent 2 mm and 100 µm. (C) Quantitation of posteriorly (P) located epithelial cells in cross sections of cataractous lenses. Error bars represent SEM. In C we used: n = 10 wildtype and n = 9 *BubR1* ^+/GTTA^ 24-month-old animals.

A second early aging-associated phenotype of *BubR1* hypomorphic mice is cataract formation [21]. MVA patients are also prone to cataracts, as well as other eye anomalies [16], [29]. Our biweekly inspections of *BubR1* ^+/GTTA^ and wildtype mice revealed that cataract formation was significantly accelerated in *BubR1* ^+/GTTA^ mice, with 50% of *BubR1* ^+/GTTA^ mice having cataracts at 101 weeks versus 116 weeks for wildtype mice (Figure 4A). Affected lenses of both *BubR1* ^+/GTTA^ and wildtype mice had nuclear cataracts, as revealed by histological evaluation (Figure 4B). Similar to *BubR1* ^H/H^ lenses [21], *BubR1* ^+/GTTA^ lenses exhibited posteriorly located epithelial cells (Figure 4B and 4C), although Morgagnian globules, which are a distinguishing feature of hypomorphic lenses, were not detected. In contrast, posterior epithelial cells were rarely observed in wildtype lenses. Taken together, the above data demonstrate that skeletal muscle degeneration and cataract formation, two hallmarks of chronological aging in humans [44], are accelerated in mice carrying the *BubR1* GTTA mutation found in human MVA syndrome.

### *BubR1* GTTA carriers exhibit early age-related fat loss

**Figure 5:**
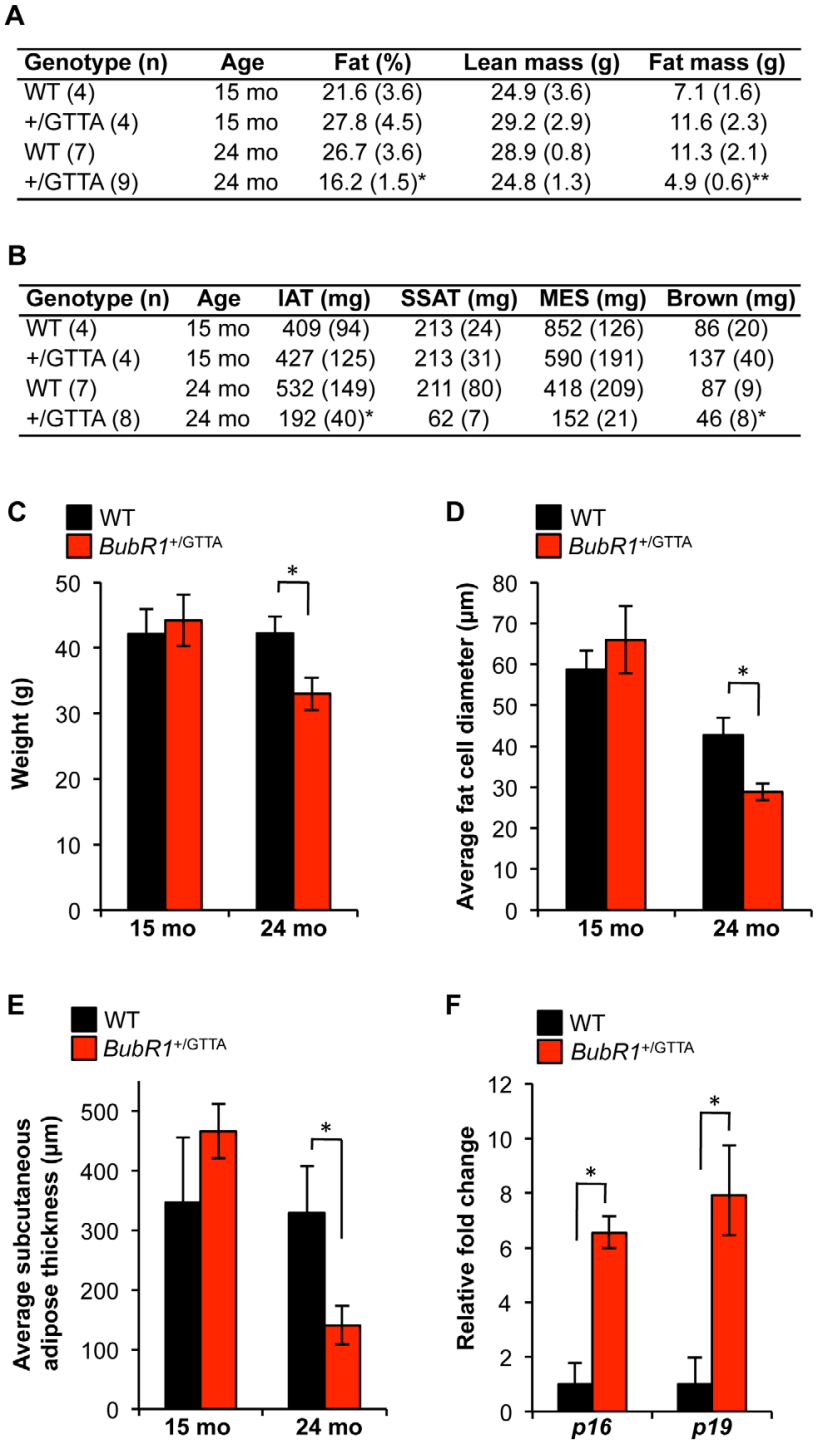
Age-related fat loss in *BubR1* ^+/GTTA^ mice. (A) Body fat percentage, lean mass and fat mass of wildtype and *BubR1* ^+/GTTA^ mice measured by DEXA scanning. (B) Fat depot sizes in wildtype and *BubR1* ^+/GTTA^ mice at the indicated ages. (C) Body weight of wildtype and *BubR1* ^+/GTTA^ mice at the indicated ages. (D) Average fat cell diameters in IAT of mice at the indicated ages. (E) Average subcutaneous adipose layer thickness of lateral skin of the indicated mice. (F) Expression of *p16* ^Ink4a^ and *p19* ^Arf^ in gastrocnemius muscles of 24 month-old mice analyzed by qRT-PCR. For all analyses error bars represent SEM. For C-F we used: n = 4 15-month-old wildtype and *BubR1* ^+/GTTA^ males; n = 7 24-month-old wildtype males; and n = 8 *BubR1* ^+/GTTA^ 24-month-old males. An unpaired t-test was used for statistical analysis in A–F: *P<0.05 and **P<0.01.

In humans, the amount of fat tissue increases during middle age but then decreases at advanced age [45]. Furthermore, during and after middle age, fat redistributes from subcutaneous to intra-abdominal visceral depots. In turn, these deposits shrink in the elderly as a result of fat redistribution to bone marrow, muscle, and liver [45]. Previous studies have demonstrated that *BubR1* ^H/H^ progeroid mice prematurely lose fat from various depots and the subdermal adipose layer [38], [46], [47]. To probe for premature changes in fat mass and redistribution in *BubR1* ^+/GTTA^ mice, we measured the overall amount of fat (using DEXA scanning on live animals), the mass of various fat deposits, the subdermal adipose thickness, and the fat cell size of 15- and 24-month-old *BubR1* ^+/GTTA^ and wildtype males. Body weight, total fat mass, and weights of major fat depots, including inguinal adipose tissue (IAT), subscapular adipose tissue (SSAT) and mesenteric adipose tissue (MES), were all normal in 15-month-old *BubR1* ^+/GTTA^ mice (Figure 5A–5C). All these values remained unchanged in 24-month-old wildtype mice. In contrast, however, body weight, percentage of body fat, and total fat mass of *BubR1* ^+/GTTA^ were all significantly reduced at 24 months. In addition, several fat depots shrank significantly, including IAT and brown fat, while SSAT and MES were trending downward.

Histological analysis showed that the average diameter of fat cells in IAT of *BubR1* ^+/GTTA^ mice declined significantly between 15 and 24 months (Figure 5D). Fat cell size also decreased in wildtype mice, but to a lesser extent than in *BubR1* ^+/GTTA^ mice. Cross sections prepared from lateral skin from 15- and 24-month-old *BubR1* ^+/GTTA^ and wildtype males revealed a dramatic decline in subdermal adipose layer thickness with aging selectively in *BubR1* ^+/GTTA^ males (Figure 5E). In contrast, dermal layer thickness did not decline (Figure S5).

Loss of fat tissue in *BubR1* ^H/H^ mice is, at least in part, due to accumulation of senescent cells [38], [47]. To determine whether accelerated fat loss in *BubR1* ^+/GTTA^ mice might involve cellular senescence, we measured *p16* ^Ink4a^ and *p19* ^Arf^ transcript levels in IAT of 24-month-old *BubR1* ^+/GTTA^ and wildtype mice by qRT-PCR. Levels of both senescence markers were markedly increased in *BubR1* ^+/GTTA^ mice (Figure 5F), indicating that early accumulation of senescent cells contributes to age-related fat loss in heterozygous carriers of the *BubR1* GTTA mutation found in MVA patients.

### *BubR1* ^+/GTTA^ mice develop large carcinogen-induced lung tumors

MVA patients are prone to tumor formation, including patients with mono-or bi-allelic *BUBR1* mutations [15], [16]. Consistent with this, *BubR1* ^H/H^ mice are highly susceptible to carcinogen-induced tumors, although it should be noted that spontaneous tumor rates are not increased [21], [23] (Table S1). Similar results have been reported for mice in which one *BubR1* allele has been inactivated [19]. As a first step to evaluate whether *BubR1* ^+/GTTA^ mice might be tumor prone we sacrificed *BubR1* ^+/GTTA^ and wildtype mice at 24 months of age and screened internal organs for overt tumors. This analysis revealed that the incidence of spontaneous tumors was similar for both genotypes (Figure S6A). Analysis of the tumor spectra revealed no significant increases in incidence of individual tumor types (Figure S6B), although there was a trend for increased tumor multiplicity in *BubR1* ^+/GTTA^ mice.

**Figure 6:**
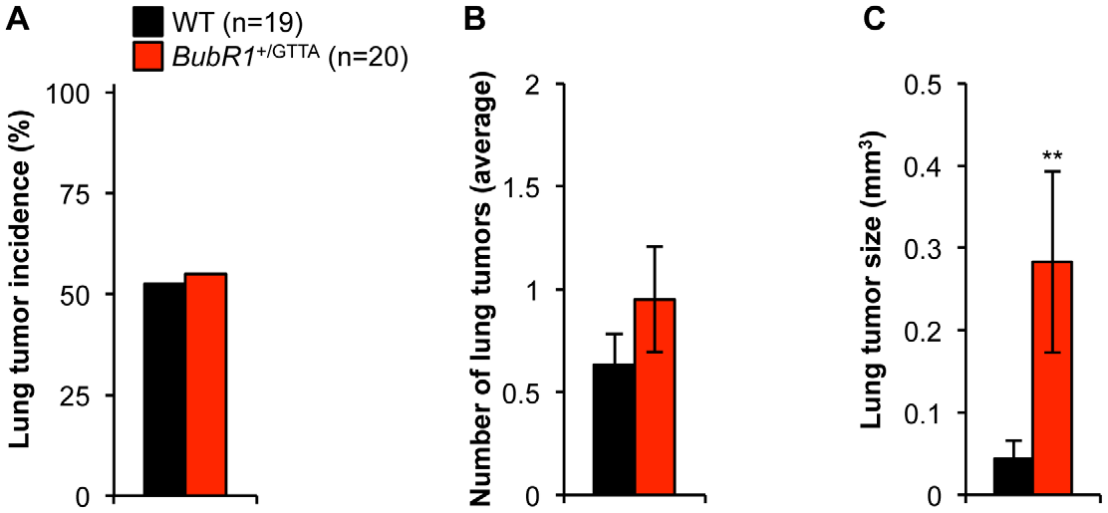
Carcinogen-induced lung tumor size is increased in *BubR1* ^+/GTTA^ mice. (A) Incidence of DMBA-induced lung tumors. (B) Lung tumor multiplicity. (C) Average lung tumor size. An unpaired t-test was utilized to determine statistical significance: **P<0.01. Color codes in B and C are as in A. For all analysis error bars represent SEM; n = 19 wildtype and n = 20 *BubR1* ^+/GTTA^ mice.

To complement these studies, we treated *BubR1* ^+/GTTA^ and wildtype mice with the carcinogen 7,12-dimethylbenz(a)anthracene (DMBA) at postnatal day 5. The mice were then sacrificed at 4 months and analyzed for lung tumor formation. We found that the tumor incidence and the tumor multiplicity were both very similar in *BubR1* ^+/GTTA^ and wildtype mice (Figure 6A and 6B). Interestingly, however, tumor size was dramatically increased in *BubR1* ^+/GTTA^ mice (Figure 6C). These findings suggest that the *BubR1* GTTA mutation has no obvious impact on tumor initiation but can promote growth of established tumors.

### Mono-allelic loss of BubR1 reduces lifespan

**Figure 7:**
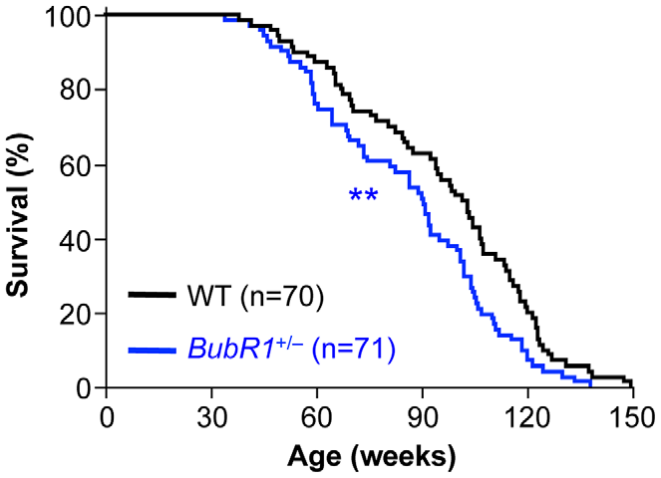
Lifespan of *BubR1* ^+/−^ mice is reduced. Kaplan-Meier overall survival curves. Asterisks denote significance compared to wildtype mice using a log-rank test. **P<0.01.

The finding that *BubR1* ^+/GTTA^ and *BubR1* ^+/−^ MEFs have similar wildtype BubR1 protein levels and the truncated protein encoded by the *GTTA* allele is expressed at non-detectable levels raised the question whether *BubR1* ^+/−^ mice might be phenotypically similar to *BubR1* ^+/GTTA^ mice. *BubR1* ^+/−^ MEFs show karyotypic similarity to *BubR1* ^+/GTTA^ MEFs in that their aneuploidy rates are also modestly increased [21]. On the other hand, PCS, a hallmark of MVA patients [15]–[18], [25]–[27], is elevated in *BubR1* ^+/GTTA^ MEFs but not in *BubR1* ^+/−^ MEFs. In an earlier study, in which survival of *BubR1* ^H/H^, *BubR1* ^+/−^, *BubR1* ^+/H^, and *BubR1* ^+/+^ mice was analyzed for up to 15 month of age, yielded no difference in survival between *BubR1* ^+/−^ and *BubR1* ^+/+^ mice [21]. However, a retrospective analysis of survival records of *BubR1* ^+/−^ and *BubR1* ^+/+^ animals that were maintained until natural death revealed that mono-allelic loss of *BubR1* significantly reduces the median lifespan (90 weeks compared to 102 weeks for the corresponding *BubR1* ^+/+^ mice, Figure 7). There was no statistically significant decrease in maximum lifespan. We note that these earlier *BubR1* ^+/−^ and *BubR1* ^+/+^ cohorts of mice were not analyzed for any age-related phenotypes (see Table S1).

## Discussion

Biallelic mutations in *WRN*, *CSA* and *CSB*, and DNA repair genes such as *XPB*, *XPD* and *TTD* are associated with human diseases that have features of premature aging [48]–[50]. MVA syndrome has some progeroid traits, but unlike the above syndromes has not been widely recognized as a progeroid disorder [17]. Whether the spectrum of age-related phenotypes of MVA patients is broader than reported has been difficult to assess, mainly because MVA patients are very rare and die early [15], [16]. It is also unknown whether parents of MVA patients are susceptible to any of the pathologies associated with MVA syndrome. We engineered a mouse model to mimic the *BubR1* nonsense mutation 2211insGTTA found in MVA patients with bi-allelic *BUBR1* mutations and show that these mice have a significantly shorter lifespan and develop several age-related disorders at accelerated rates, including sarcopenia, cataracts, and loss of fat tissue. These findings strengthen the notion that MVA syndrome is a progeroid syndrome, and provide important experimental evidence for the longstanding concept that variations in select genes may affect the rate of age-related deterioration in certain tissues and organs.

To our knowledge, accelerated age-related pathologies have not been reported in parents of affected individuals with any of the classical recessive human progeroid syndromes [48]–[51]. Furthermore, while homozygous knockout or mutant mice have been established for most of the implicated genes, including *WRN*, *CSA* and *CSB*, *XPB*, *XPD*, and *TTD*, whether heterozygotes of these models have reduced longevity or a faster than normal onset of age-related functional decline in particular tissues has not been studied in detail [50], [52]–[56]. Thus, based on the findings presented here, it will not only be important to determine whether heterozygous MVA *BubR1* mutations other than 2211insGTTA cause age-related phenotypes in mice, but also to perform similar studies on mice heterozygous for other progeria-associated genes. Three observations suggest that the phenotypes of *BubR1* ^+/GTTA^ mice are likely to be caused by reduced expression of wildtype BubR1 protein without a contribution of truncated BubR1 protein. First, the truncated protein encoded by the *GTTA* allele is non-detectable by western blotting, indicating that its level of expression is very low. Furthermore, residual levels of wildtype BubR1 protein are similar in *BubR1* ^+/GTTA^ and *BubR1* ^+/−^ MEFs. Second, *BubR1* ^+/GTTA^ and *BubR1* ^+/−^ MEFs have similar aneuploidy rates, suggesting that the extent to which BubR1 is dysfunctional is the same. Third, *BubR1* ^+/GTTA^ and *BubR1* ^+/−^ mice show very similar reductions in median lifespan. It will be important to complement the survival data of *BubR1* ^+/−^ mice with a comprehensive analysis of age-related phenotypes, and new cohorts of *BubR1* ^+/−^ and *BubR1* ^+/+^ mice are currently being established for this purpose.

Previously, we have shown that clearance of p16^Ink4a^-positive senescent cells from *BubR1* ^H/H^ mice results in attenuation of sarcopenia, fat loss, and cataracts, indicating that accumulation of senescent cells in skeletal muscle, adipose tissue, and eye drives functional decline in these tissues [38]. This, combined with the observation that *p16* ^Ink4a^ and *p19* ^Arf^ transcript levels are elevated in skeletal muscle and fat of *BubR1* ^+/GTTA^ mice suggests that senescence contributes to the accelerated functional decline in these animals. On the other hand, accelerated cataractogenesis in *BubR1* ^+/GTTA^ mice seems to be senescent cell independent (data not shown). Perhaps, the mere accumulation of epithelial cells in the posterior portion of the lens is sufficient to accelerate cataract formation. The main difference between *BubR1* ^+/GTTA^ and *BubR1* ^H/H^ lenses is that the latter have posteriorly located Morgagnian globules [21], which may be associated with senescence and explain why cataractogenesis is much more accelerated in *BubR1* ^H/H^ than in *BubR1* ^+/GTTA^ mice.

The mechanism by which BubR1 insufficiency induces senescence appears to be more complicated than anticipated [21], [23], [57]. It is unlikely that aneuploidy represents the primary lesion that drives senescence, mainly because other aneuploidy models with substantially higher aneuploidy rates do not undergo premature senescence and aging [1], [4], [6], [58]. BubR1 is expressed in interphase where it apparently continues to serve as an inhibitor of APC/C^Cdc20^ activity [37]. Consistent with this, recent reports indicate that APC/C^Cdc20^ E3 ubiquitin ligase activity orchestrates key developmental processes in post-mitotic neurons, including dendrite growth and presynaptic differentiation [59], [60]. These findings raise the interesting possibility that BubR1 insufficiency might lead to unscheduled degradation of APC/C^Cdc20^ substrates in interphase cells, which, in turn, could lead to cellular stresses that engage p16^Ink4a^ and induce senescence. Key progeroid phenotypes of *BubR1* ^H/H^ mice are also observed in *BubR1* ^+/GTTA^, but are considerably milder, which correlates with less profound BubR1 protein insufficiency (Table S1). Various phenotypes seem unique to *BubR1* ^H/H^ mice including cardiac dysfunction, dwarfism, facial dysmorphisms, and thinning of the dermis, suggesting that a more extreme level of BubR1 insufficiency is required for their induction.

*BubR1* ^+/GTTA^ mice are not prone to spontaneous tumors and show normal tumor incidence and multiplicity when challenged with the carcinogen DMBA. The most straightforward explanation would be that the level of aneuploidization is insufficient to promote neoplastic transformation. Consistent with this, *Bub3* ^+/−^ mice have similarly mild aneuploidy rates as *BubR1* ^+/GTTA^ mice and are also not prone to spontaneous or DMBA-induced tumors [61]. However, a significant feature of DBMA-induced lung tumors of *BubR1* ^+/GTTA^ mice is their large size, indicating that the mutation promotes tumor aggressiveness without impacting tumor initiation. It will be interesting to determine whether individuals carrying the 2211insGTTA mutation are prone to lethal malignancies.

Our current study and previous data support the notion that BubR1 protein levels tightly correlate with aneuploidy rates, cancer susceptibility, lifespan and aging-related phenotypes (Table S1), indicating that BubR1 is a key determinant of healthspan and lifespan and warrants a comprehensive analysis of the health status of parents of MVA patients and relatives that are also heterozygous carriers of the same MVA *BUBR1* mutations. In addition, it would be interesting to screen for *BUBR1* mutations in the general population, either in an unbiased manner or more selectively in cohorts prone to conditions associated with BubR1 insufficiency in mice, including sarcopenia, cataracts and fat tissue dysfunction. Subsequent characterization of these mutations, for instance for impact on BubR1 protein stability, might lead to the identification of additional *BUBR1* variants that influence rates of age-related deterioration in certain tissues and organs.

## Materials and Methods

### Generation of *BubR1* ^+/GTTA^ mice

The *BubR1* ^GTTA^ allele was produced by a recombineering based approach [62]. Briefly, a genomic *BubR1* gene fragment of 10 kb spanning exons 14–19 was retrieved from BAC #bMQ_294E2 (129S7/SvEv ES Cell, Source BioScience) and transferred into pDTA. Insertion of the GTTA sequence into the exon 17 (c.2178_2179) was done as follows: a tetracyclin-resistance gene cassette was made by PCR using pKOEZ-40 plasmid as a template (gift from Dr. Pumin Zhang), the tetra-partite forward primer (50 bp homology to the target region, GTTA, *Cla*I, 24 bp homology to the Tet) 5′-CCTGG TGTTCACAGTATCGCCTACAACTGTTAAAATCCCTACTAGAATTAGTTAATCGATGGTCGA CGGTATCGATAAGCTTGA-3′ and the tri-partite reverse primer (50 bp homology to the target region, *Cla*I, 24 bp homology to the Tet) 5′-TCCAGCACAGGCATCGGTC GGTCTTCCACAGAAAACTCCGCAAAAGCACTATCGATTTGGATGGTGAATCC GTTAGCGA-3′. The GTTA-*Cla*I-Tet-*Cla*I cassette was inserted into the pDTA-*BubR1*(E14-E19) by recombineering. The resulting construct was digested with *Cla*I and re-ligated for removal of the Tet gene. Next, a loxP-neomycin phosphotransferase II (neo) gene-loxP cassette was inserted into the pDTA-*BubR1* ^GTTA^ (E14-E19) construct, 141 bp upstream of exon 17, using recombineering [62]. The final targeting vector was linearized with *Mlu*I and electroporated into TL1 129Sv/E ES cells. Transfectants were selected in 350 µg/ml G418 and 0.2 µM FIAU, and expanded for Southern blot analysis using a 1155 bp 5′ external probe on *Xmn*I/*Xho*I -digested genomic ES cell DNA. The probe was amplified by PCR from 129Sv/E genomic DNA using the following primers: 5′-GCAGAGTATCCTGACAGGTTAAGGCAC-3′ and 5′-CATAATAATTATCCAACCATGAATGATC-3′. Chimeric mice were produced by microinjection of three independent ES cell targeted clones with 40 chromosomes into C57BL/6 blastocysts. Chimeric males were mated with C57BL/6 females and germline transmission of the *BubR1* ^NEO;GTTA^ allele was verified by PCR analysis of tail DNA from pups with a agouti coat color. By crossing *BubR1* ^+/NEO;GTTA^ mice to protamine-Cre transgenics [63] the NEO cassette was excised. The following primer combinations were used for PCR genotyping of mice used in our studies: primers a (5′-TCAGATCTCCTAGAGCTGGGGTTA-3′) and b (5′-AATTCTAGTAGGGATTTTAA CAGTTG-3′) for *BubR1* ^GTTA^; primers c (5′-GTCTTGTCGATCAGGATGATCTG-3′) and d (5′-GAAGTAGTATTGTTCCTGTGG CAT-3′) for *BubR1* ^NEO;GTTA^. The targeting vector, targeted ES cells as well as *BubR1* ^NEO;GTTA^ mice were sequenced for the presence of GTTA insertion by PCR amplifying the 1.1 kb fragment using primers c and d. Note: in humans, 2211insGTTA results into S738fsX753 (see Mutation 1, shared by Family 2 and Family 3 [15]). In mice, 2178–9insGTTA results in S727fsX750. Insertion of GTTA followed by *Cla*I coding sequence, as in our targeting strategy, leads to S727fsX730. All mice, including *BubR1* ^+/−^ and *BubR1* ^+/+^ mice used for survival analysis, were on a mixed 129 X C57BL/6 genetic background and housed in a pathogen-free barrier and maintained on a 12 hours dark/light cycle throughout the study. Mice had ad libitum access to food containing 10% fat and were inspected daily. Animals used for survival analysis were mice found dead or sacrificed when moribund. Mice sacrificed at 15 and 24-months of age were screened for lymphomas, carcinomas and sarcomas to assess spontaneous tumorigenesis. Animals sacrificed at 3, 15 and 24-months for analyses were omitted from survival, lordokyphosis and cataract incidence curves. All animal protocols were reviewed and approved by the Mayo Clinic institutional animal care and use committee.

### Generation and culture of MEFs

Wildtype and *BubR1* ^+/GTTA^ MEFs were generated and cultured as previously described [46]. MEFs were frozen at passage 2 or 3 and used for experimentation at the indicated passages. At least three wildtype and *BubR1* ^+/GTTA^ lines were used for all experiments. Mitotic MEFs were generated as previously described [36].

### Western blotting

Western blot analysis was performed as previously described [64]. Tissue lysates were prepared by first snap freezing the tissue in liquid nitrogen upon sacrifice. Frozen tissue was ground into fine powder with pestle and mortar. 20 mg of tissue powder was suspended in 200 µl lysis buffer (0.1% NP-40, 10% glycerol in PBS, plus protease inhibitors) and vortexed for 10 min at 4°C. After centrifugation at 14000 rpm at 4°C, 150 µl supernatant was transferred to a 0.5 ml PCR tube and 150 µl Laemmli lysis buffer was added. The lysate was boiled for 10 min before loading on Tris-HCl Polyacrylamide gel. Blots were probed with antibodies for BubR1 (BD), pH3^Ser10^ (Millipore) and ß-actin (Sigma). Ponceau S stain was used as a loading control. Quantification of BubR1 protein levels in *BubR1* ^+/GTTA^ and *BubR1* ^+/−^ MEF lysates was done as described [65].

### Karyotype analysis

Karyotype analysis on P5 wildtype and *BubR1* ^+/GTTA^ MEFs was performed as described [66].

### Analysis of age-related phenotypes

Bi-weekly monitoring for lordokyphosis and cataract incidence was performed as described [23]. Fiber diameter measurements were performed on cross sections of the gastrocnemius and abdominal muscle from 3 and 15-month-old mice according to previously described methods [23]. Dissection of the paraspinal muscle was performed halfway between the front and hind limb, and processed and measured as the other skeletal muscles. The mean was calculated from a total of fifty measurements obtained with a calibrated program (Olympus MicroSuite Five). Measurements of fat cell diameters were performed according to the same method. Measurements of the total thickness of dermis and subcutaneous adipose layer of lateral skin were performed as described [46]. For histological evaluation of cataracts, whole eyes were paraffin embedded, sagittally sectioned through the middle of the lens and stained with hematoxylin and eosin. The number of cells that had migrated past the epithelial bow of the lens was counted as posterior localized epithelial cells. DEXA scanning was used to measure bone mineral density, bone mineral content, percentage of total body fat, lean mass and fat mass. These measurements were done as described [67]. Treadmill exercise tests were performed as described [43]. For isoproterenol stress tests, a lethal dose of isoproterenol (680 mg/kg) was injected in the chest cavity and time to death was recorded. Mice were monitored for cardiac arrhythmias using a Vevo2100 ultrasound system (Visualsonics) as described [68].

### Quantitative RT–PCR

qRT-PCR analysis on cDNA derived from RNA isolated from various mouse tissues was as described [23].

### Tumor susceptibility

Mice were tested for DMBA induced tumor formation as previously described [69].

## Supporting Information

Figure S1 Quantification of BubR1 protein levels in *BubR1* ^+/GTTA^ and *BubR1* ^+/−^MEFs. (A) Western blot analysis of serially diluted wildtype and representative *BubR1* ^+/GTTA^ and *BubR1* ^+/−^ MEF lysates probed for BubR1. Actin was used as a loading control. (B) The average BubR1 signal intensity of three independent wildtype MEF lines plotted against percentage of lysate volume loaded using the equation (Top left). (C) Relative BubR1 protein amount in 3 independent *BubR1* ^+/GTTA^ and *BubR1* ^+/−^ MEF lines presented as average ± SD.
(TIF)

Figure S2 Heart function appears normal in *BubR1* ^+/GTTA^ mice. (A) Cardiac arrhythmia measurements of wildtype and *BubR1* ^+/GTTA^ 15-month-old male mice shown as percentage of sinus pause disturbances per heartbeat. BPM, beats per minute. (B) Cardiac stress tolerance was determined by injection of a lethal dose of isoproterenol (680 mg/kg) and time to death was recorded. Error bars represent SEM. For isoproterenol experiments 4 wildtype and 4 *BubR1* ^+/GTTA^ 15-month-old males were used.
(TIF)

Figure S3 *BubR1* ^+/GTTA^ mice show no evidence of sarcopenia at a young age. Mean fiber diameter measurements on cross sections of the gastrocnemius (Gastro), abdominal (ABD) and paraspinal muscle in wildtype and *BubR1* ^+/GTTA^ mice at 3 months of age. Error bars represent SEM. For all analysis n = 3 wildtype and n = 5 *BubR1* ^+/GTTA^ males.
(TIF)

Figure S4 Bone composition is similar in aged *BubR1* ^+/GTTA^ mice. Bone composition of 15-month-old wildtype and *BubR1* ^+/GTTA^ mice as determined by DEXA scanning. BMD, bone mineral density; BMC, bone mineral content.
(TIF)

Figure S5 Normal age-related decline of dermal thickness in *BubR1* ^+/GTTA^ mice. Measurements of the average dermal thickness on cross sections from the lateral skin at the indicated ages. Error bars represent SEM. n = 4 15-month-old wildtype and *BubR1* ^+/GTTA^ males; n = 7 24-month-old wildtype mice; and n = 8 *BubR1* ^+/GTTA^ 24-month-old mice.
(TIF)

Figure S6 *BubR1* ^+/GTTA^ mice are not prone to spontaneous tumors. (A) Spontaneous tumor incidence in 24-month-old wildtype and *BubR1* ^+/GTTA^ animals. Mice were sacrificed and screened for lymphomas, carcinomas and sarcomas.(B) Tumor spectrum of 24-month-old wildtype and *BubR1* ^+/GTTA^ mice. We note that values in A and B were not statistically different (Fishers' exact test).
(TIF)

Table S1 Summary of aging-related phenotypes in wildtype, *BubR1* ^+/GTTA^, *BubR1* ^+/−^ and *BubR1* ^H/H^ mice. Aneuploidy rates in MEFs are indicated as percentage increase over wildtype controls (wildtype controls for BubR1+/GTTA MEFs had 12% aneuploidy, whereas those of *BubR1* ^+/−^ and *BubR1* ^H/H^ MEFs had 9% aneuploidy). ND, not determined. ^1^ denotes previous published results [21]; ^2^ [38]; and ^3^ [42].
(TIF)
