# Supplementary figures and images for: Reduced Life- and Healthspan in Mice Carrying a Mono-Allelic BubR1 MVA Mutation
Source: PLoS Genet. 2012 Dec 27;8(12):e1003138. doi: 10.1371/journal.pgen.1003138 (PMC3531486; doi:10.1371/journal.pgen.1003138)

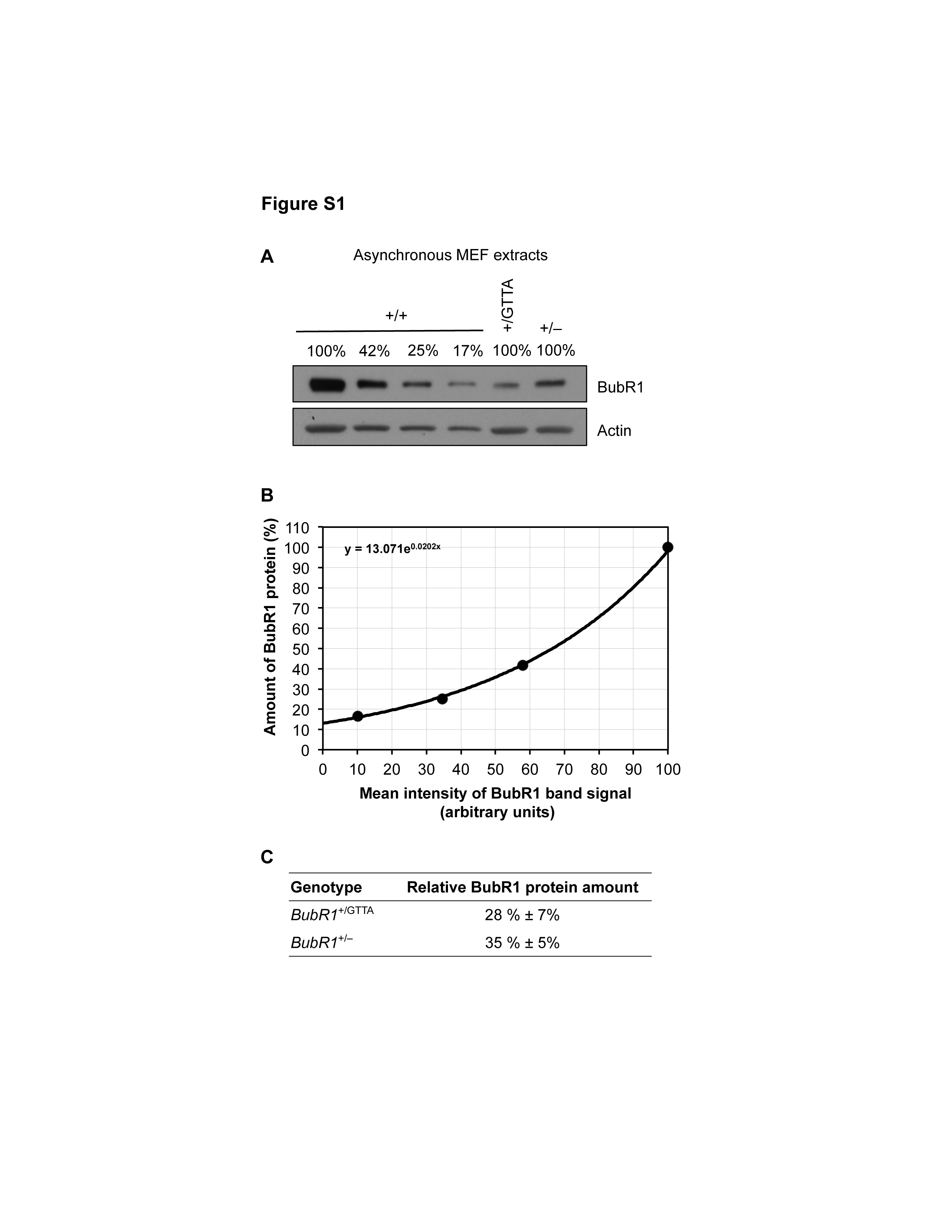

Supplement: Figure S1 — Quantification of BubR1 protein levels in BubR1 +/GTTA and BubR1 +/−MEFs. (A) Western blot analysis of serially diluted wildtype and representative BubR1 +/GTTA and BubR1 +/− MEF lysates probed for BubR1. Actin was used as a loading control. (B) The average BubR1 signal intensity of three independent wildtype MEF lines plotted against percentage of lysate volume loaded using the equation (Top left). (C) Relative BubR1 protein amount in 3 independent BubR1 +/GTTA and BubR1 +/− MEF lines presented as average ± SD. (TIF) [file pgen.1003138.s001.tif]

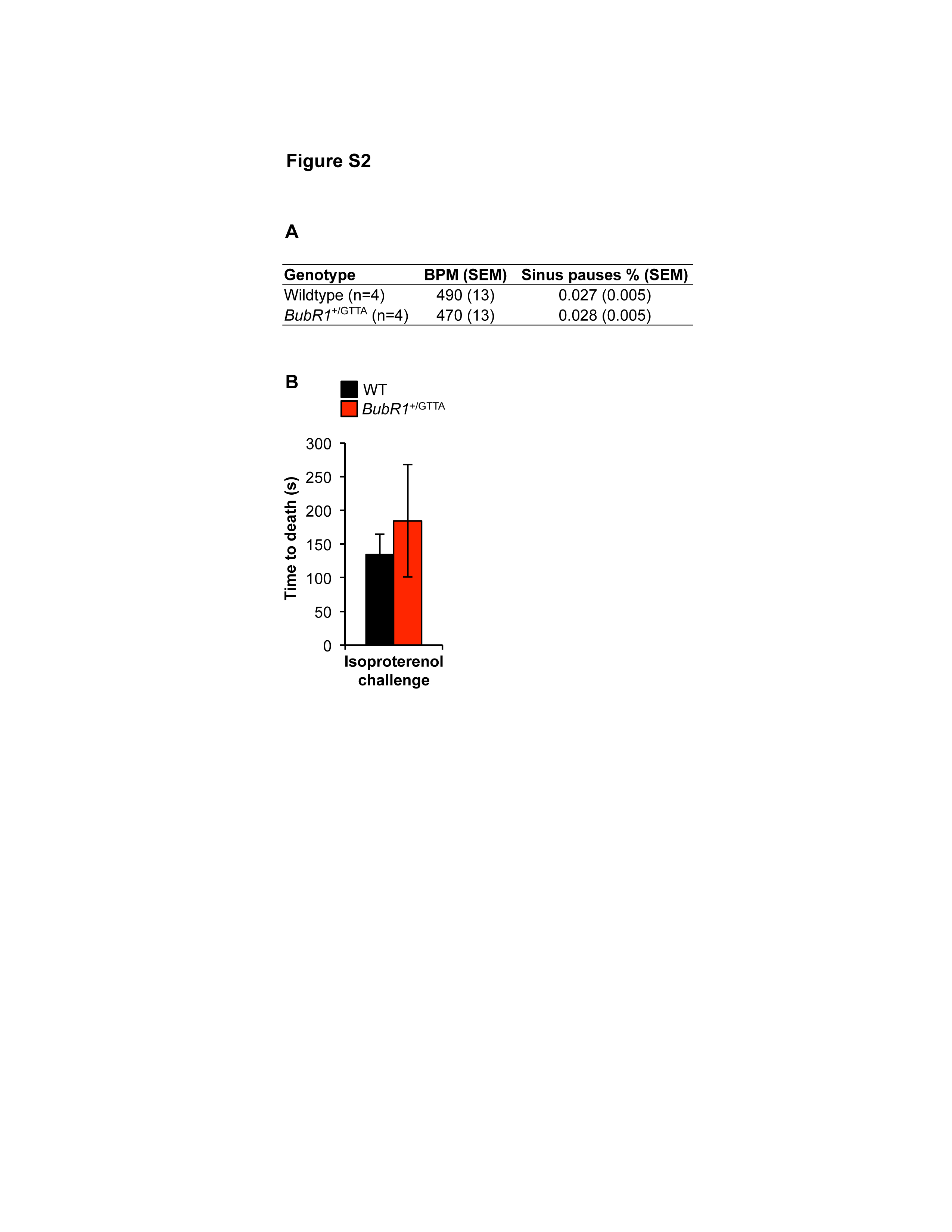

Supplement: Figure S2 — Heart function appears normal in BubR1 +/GTTA mice. (A) Cardiac arrhythmia measurements of wildtype and BubR1 +/GTTA 15-month-old male mice shown as percentage of sinus pause disturbances per heartbeat. BPM, beats per minute. (B) Cardiac stress tolerance was determined by injection of a lethal dose of isoproterenol (680 mg/kg) and time to death was recorded. Error bars represent SEM. For isoproterenol experiments 4 wildtype and 4 BubR1 +/GTTA 15-month-old males were used. (TIF) [file pgen.1003138.s002.tif]

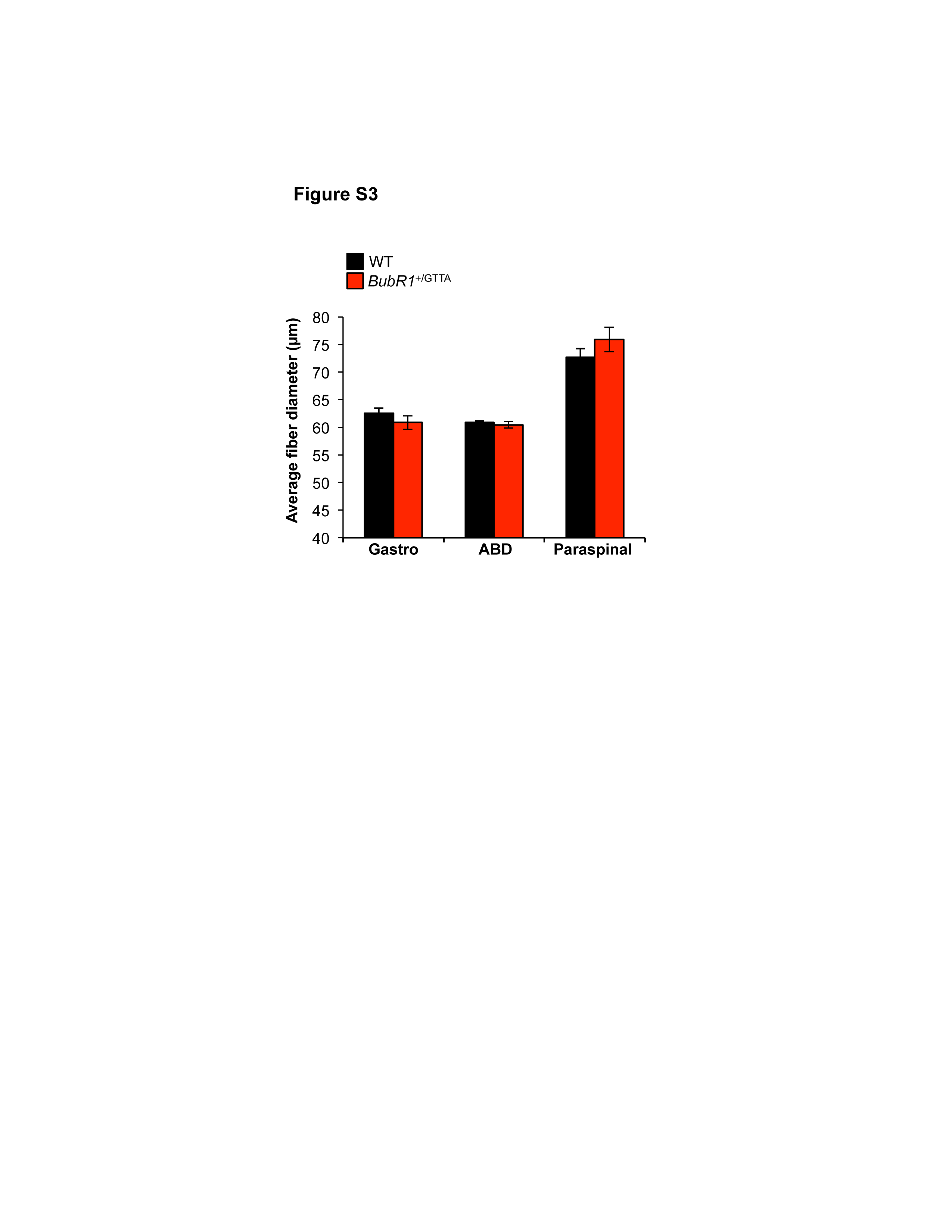

Supplement: Figure S3 — BubR1 +/GTTA mice show no evidence of sarcopenia at a young age. Mean fiber diameter measurements on cross sections of the gastrocnemius (Gastro), abdominal (ABD) and paraspinal muscle in wildtype and BubR1 +/GTTA mice at 3 months of age. Error bars represent SEM. For all analysis n = 3 wildtype and n = 5 BubR1 +/GTTA males. (TIF) [file pgen.1003138.s003.tif]

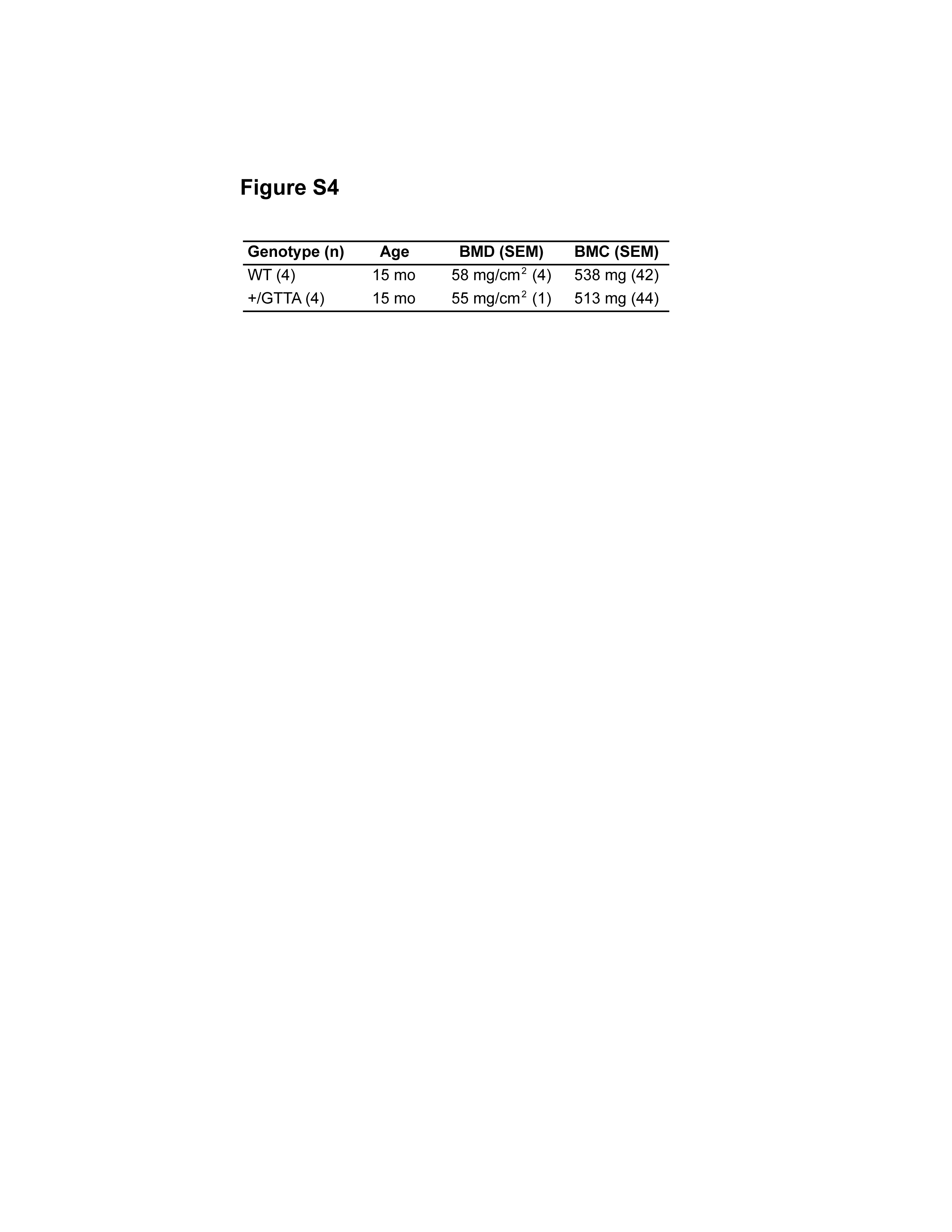

Supplement: Figure S4 — Bone composition is similar in aged BubR1 +/GTTA mice. Bone composition of 15-month-old wildtype and BubR1 +/GTTA mice as determined by DEXA scanning. BMD, bone mineral density; BMC, bone mineral content. (TIF) [file pgen.1003138.s004.tif]

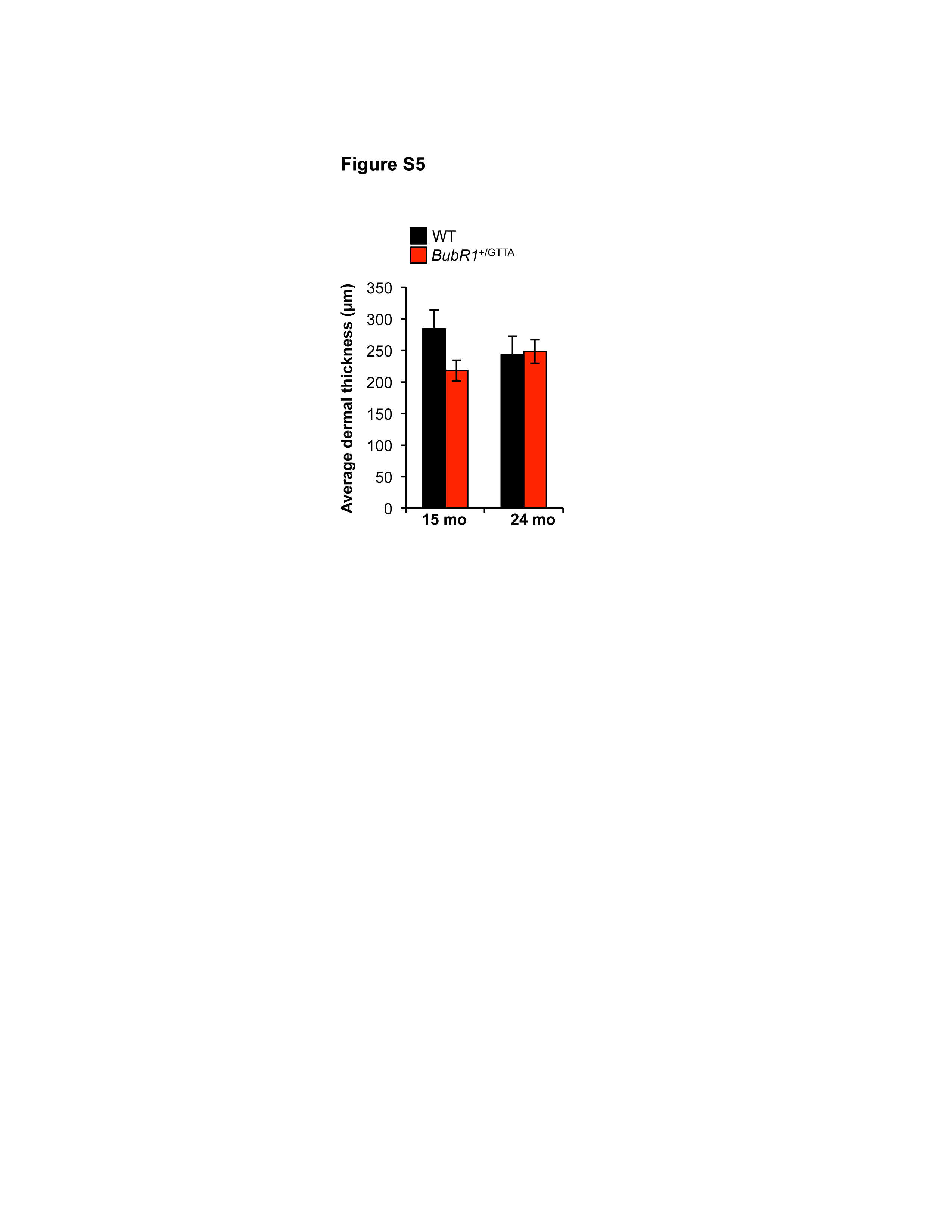

Supplement: Figure S5 — Normal age-related decline of dermal thickness in BubR1 +/GTTA mice. Measurements of the average dermal thickness on cross sections from the lateral skin at the indicated ages. Error bars represent SEM. n = 4 15-month-old wildtype and BubR1 +/GTTA males; n = 7 24-month-old wildtype mice; and n = 8 BubR1 +/GTTA 24-month-old mice. (TIF) [file pgen.1003138.s005.tif]

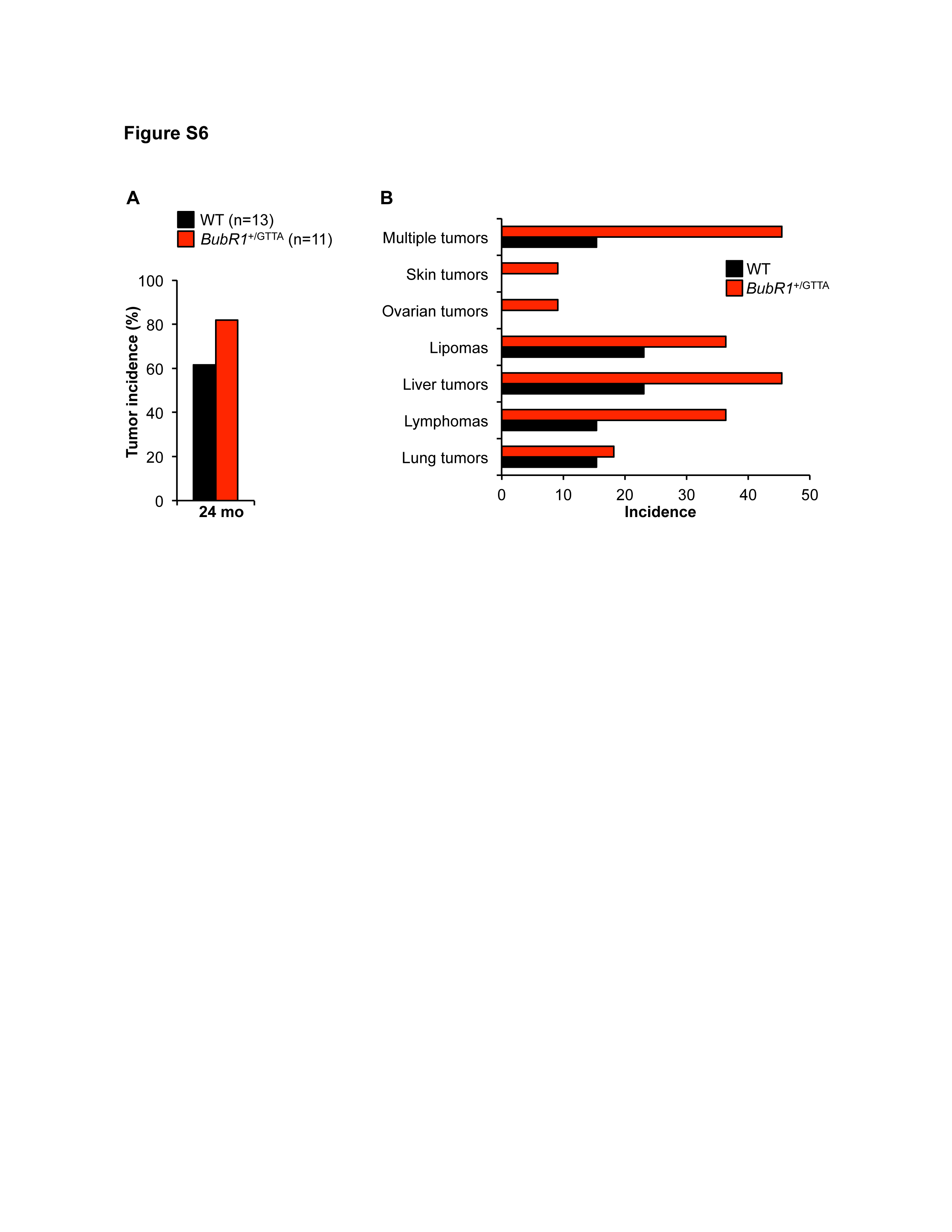

Supplement: Figure S6 — BubR1 +/GTTA mice are not prone to spontaneous tumors. (A) Spontaneous tumor incidence in 24-month-old wildtype and BubR1 +/GTTA animals. Mice were sacrificed and screened for lymphomas, carcinomas and sarcomas.(B) Tumor spectrum of 24-month-old wildtype and BubR1 +/GTTA mice. We note that values in A and B were not statistically different (Fishers' exact test). (TIF) [file pgen.1003138.s006.tif]

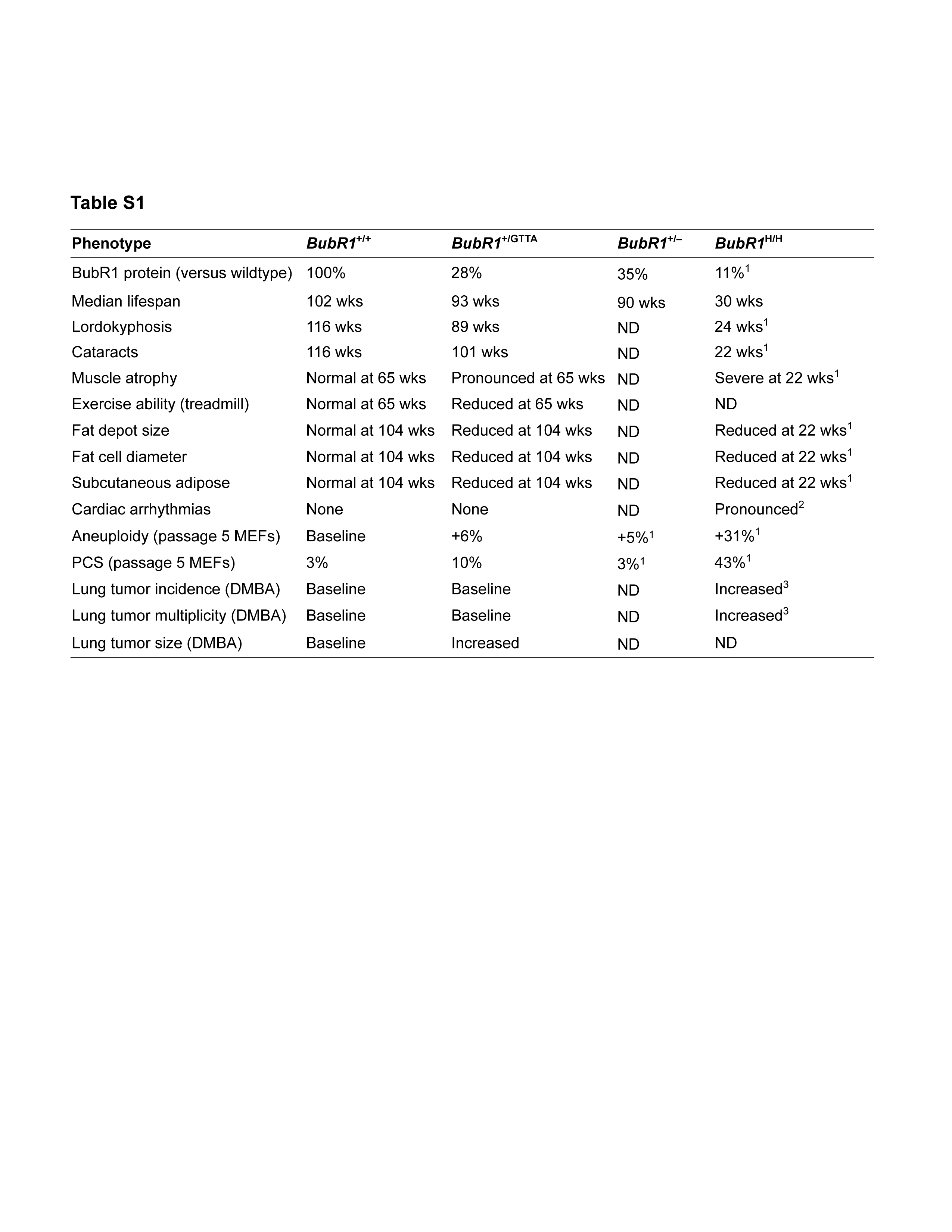

Supplement: Table S1 — Summary of aging-related phenotypes in wildtype, BubR1 +/GTTA, BubR1 +/− and BubR1 H/H mice. Aneuploidy rates in MEFs are indicated as percentage increase over wildtype controls (wildtype controls for BubR1+/GTTA MEFs had 12% aneuploidy, whereas those of BubR1 +/− and BubR1 H/H MEFs had 9% aneuploidy). ND, not determined. 1 denotes previous published results [21]; 2 [38]; and 3 [42]. (TIF) [file pgen.1003138.s007.tif]
